# Supplementary material for: Reaction nanoscopy of ion emission from sub-wavelength propanediol droplets
Source: Nanophotonics. 2023 Apr 6;12(10):1823–31. doi: 10.1515/nanoph-2022-0714 (PMC11501279; doi:10.1515/nanoph-2022-0714)
Supplement: Supplementary file 1 — Supplementary Material Details [file j_nanoph-2022-0714_suppl_001.pdf]

Supplementary Information to

Reaction Nanoscopy of Ion Emission from Sub-wavelength Propanediol Droplets

Philipp Rosenberger et al.

March 19, 2023

1 Simulation geometry

The interface between the nanodroplet and vacuum has been modeled using a slab of molecules in the x-z plane consisting of six layers as shown in Figure S1. For an unambiguous definition of the droplet-vacuum interface, the bottom three layers are a rotated image of the top layers. The formation enthalpy for the 1,2-PDO and 1,3-PDO slab from isolated gaseous molecule is -16.14 kcal/mol (0.7 eV) and -31.14 kcal/mol (1.35 eV), respectively. This suggests that simulation configurations are energetically stable.

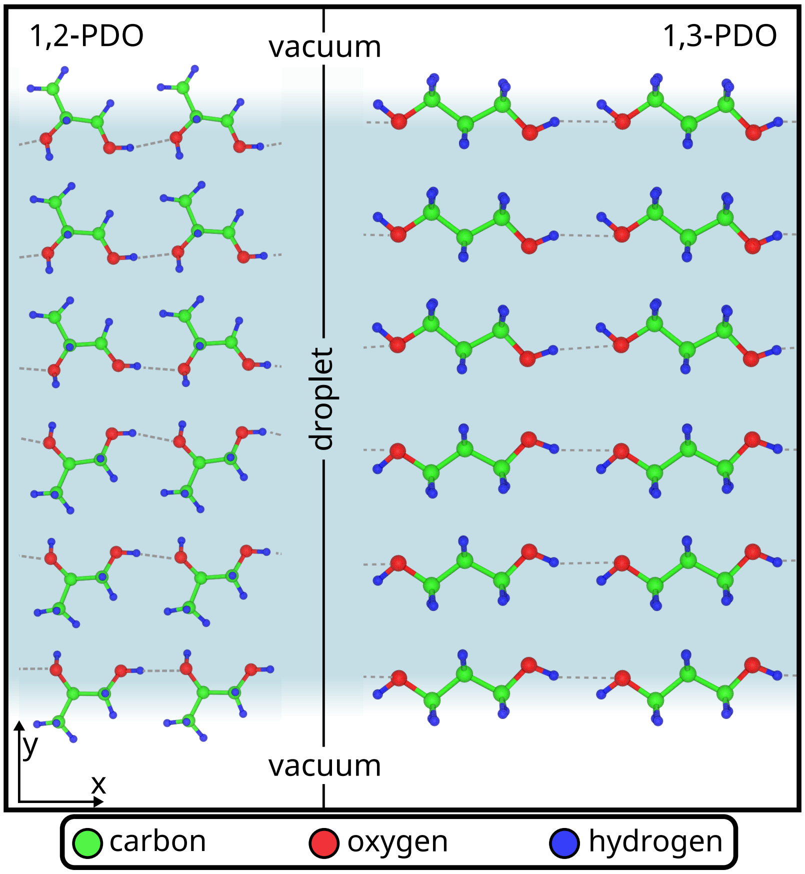

Figure S1: Side view of the simulation geometry for 1,2-PDO (left) 1,3-PDO (right). The surface normal is along the y-axis. Periodic boundary conditions were applied along x and z. The dotted lines represent hydrogen bonds.

2 Ion fragment selection

The measurement of the electron signal in reaction nanoscopy enables a significant reduction of the single molecule ionization signal in the droplet ion data. As shown in Figure 1 of the main text, droplet events cause a much larger electron signal than gas-phase events, which allows filtering the data. Despite the filtering, we always observe some contribution of gas-phase, single molecule ionization to the droplet data. From a comparison of the gas-phase data and the droplet data presented in Figure S2, it becomes clear that the peaks close to $y = 0$ in the droplet data (which show up identically in the gas-phase data) are ions emitted from single gas-phase molecules which were ionized independently from the droplet but by the same laser pulse. For this case of simultaneous ionization of a droplet and single-molecules, the electron signal is still large and the whole laser shot is assigned to the droplet data. This effect is further confirmed by the 1D time-of-flight histograms in Figure S3, where data for $|y| < 11\,\mathrm{mm}$ is shown, corresponding to the white-shaded regions of Figure 2 in the main text and Figure S2.

Similar but fragment-specific filters were applied for the ion data shown in Figure 5 of the main text. For every ion species under investigation, we defined a polygon in the space of time-of-flight and y-position. These polygons are shown in Figure S2. For the droplet data, all the polygons were additionally mirrored around $y = 0$ in order to include both lobes of the dipolar ion emission pattern.

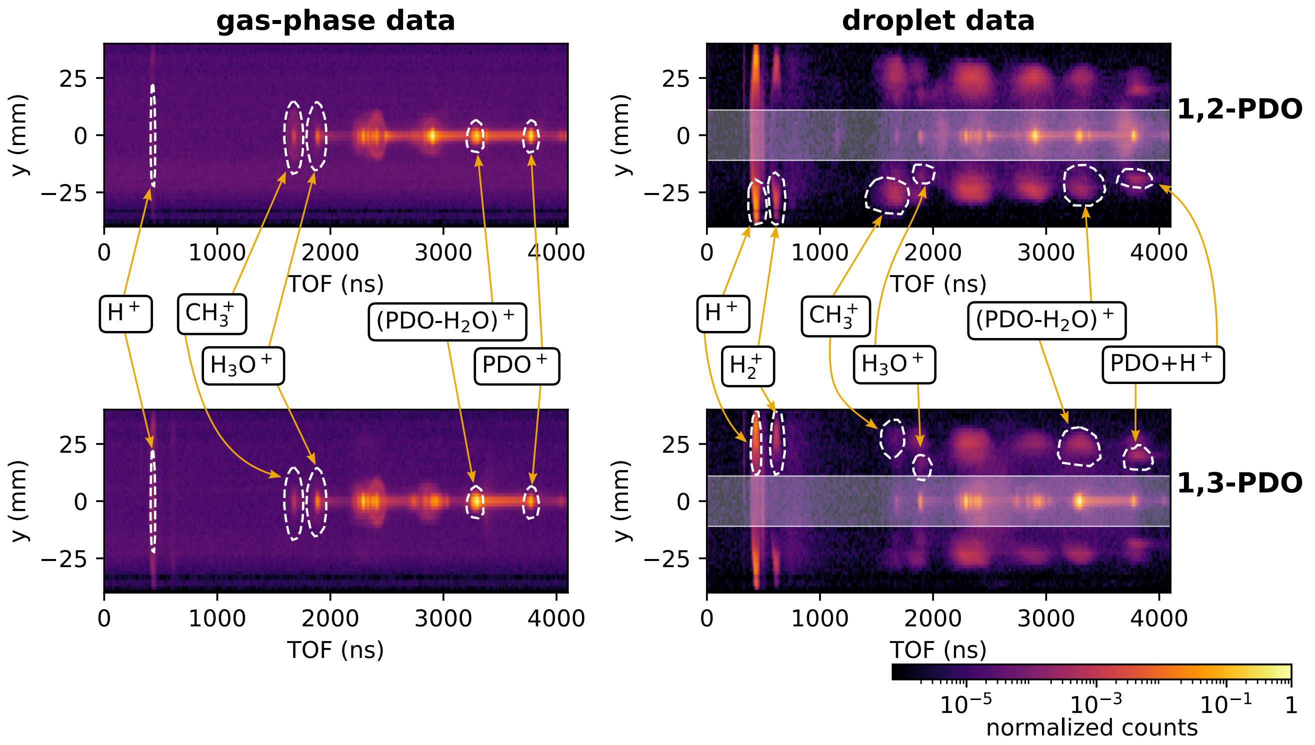

Figure S2: Filtering ion signals for gas-phase and droplet data. Two measurements are shown, one for 1,2-propanediol (1,2-PDO, panels a and b) and one for 1,3-propanediol (1,3-PDO, panels c and d). Both measurements were split into gas-phase data and droplet based on the strength of the electron signal which is recorded in coincidence with the ions. Gas-phase data (first column, panels **a** and **c**) are defined by a small or entirely absent electron signal. Droplet data (second column, panels **b** and **d**) are defined as the complementary set. The ion species listed in the middle were identified after calibrating the time-of-flight. The polygons indicated by the white dashed lines were used for the yield comparison in the main text (Figure 5). The white shading corresponds to $|y| < 11\,\mathrm{mm}$.

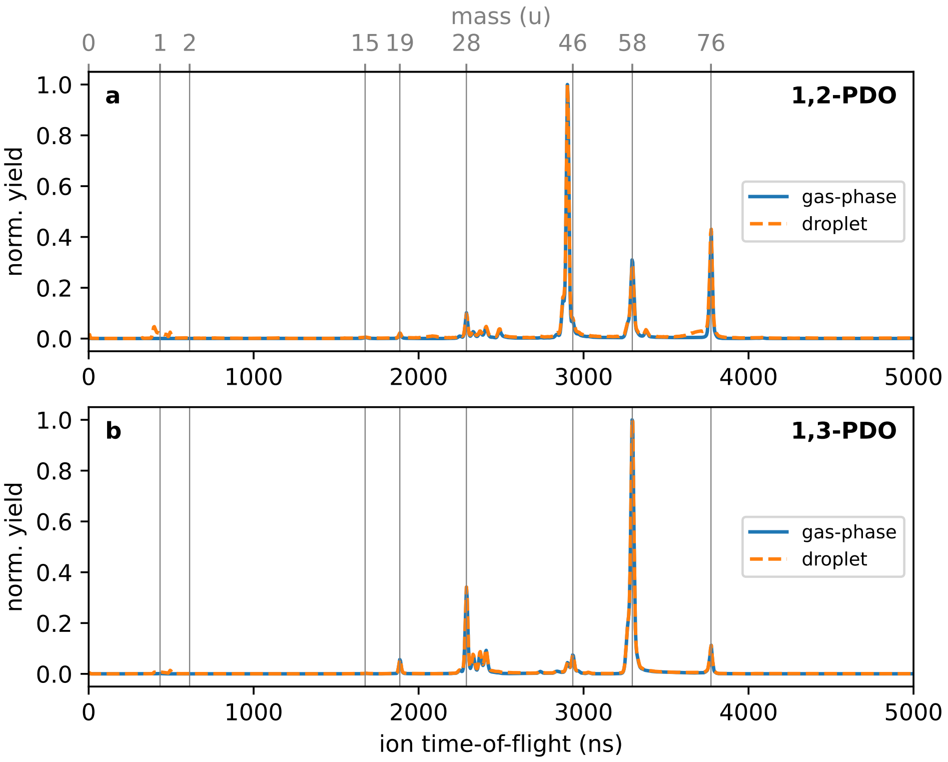

Figure S3: Time-of-flight histograms for $|y| < 11\,\mathrm{mm}$. The histograms were obtained by projecting the corresponding parts of the position-resolved spectra of Figure S2 (for the droplet data, we have indicated $|y| < 11\,\mathrm{mm}$ with the white shading).

3 Measured proton yield and relative density of large droplets

Due to the count rate limitations of delay-line detectors, the high intensity data at of Figure 3, i.e., panels c and d may not contain all protons that were produced during the measurements. However, we will show here that despite a potential saturation of the ion detector, it is still possible to make statements about the relative frequency of smaller and larger clusters in the aerosol stream.

We denote the total measured proton yield from a certain droplet size s as Y_s, the number density of such droplets as n_s and the maximum field enhancement factor as α_s. We assume that the proton yield depends on the intensity according to a power law: $Y \propto I^N$, where N is the nonlinearity of the process. The focal volume is described by a two-dimensional Gaussian, assuming an approximately cylinder-symmetric focal volume (which means that the Rayleigh range is significantly longer than the radius of the droplet stream). Lastly, we denote the intensity at which detector saturation sets in by I_{sat}.

With these definitions, the focal volume averaged measured ion yield is given by:

$$Y_s = \sigma n_s \int_{r_{\mathrm{sat}}}^{\infty} \mathrm{d}r\, r I^N(r) = \sigma n_s \alpha_s^{2N} I_0^N \int_{r_{\mathrm{sat}}}^{\infty} \mathrm{d}r\, r \exp(-Nr^2) = \frac{\sigma n_s \alpha_s^{2N} I_0^N}{2N} \exp(-N r_{\mathrm{sat}}^2). \tag{1}$$

Here, σ was introduced as a kind of nonlinear cross-section for the proton generation and the radius r_{sat} can be determined from the saturation intensity using the Gaussian shape of the focal volume:

$$r_{\mathrm{sat}} = \sqrt{\ln \frac{I_0 \alpha_s^2}{I_{\mathrm{sat}}}}. \tag{2}$$

Note that r_{sat} depends on the field enhancement factor and thus on the droplet size. Inserting this expression into Equation (1), we obtain:

$$Y_s = \frac{\sigma I_{\mathrm{sat}}^N}{2N} n_s. \tag{3}$$

Thus, despite saturation beyond I_{sat}, the measured yield Y_s from droplets of a certain size is proportional to their density in the aerosol stream n_s. All the other parameters are independent of the droplet size.

In the experiment, we can distinguish between yields from small and large droplets, Y_S and Y_L, based on the emission direction of the ions. The relative yield between large droplets L and small droplets S can be determined by taking the ratio between the number of protons emitted along the propagation direction and along the polarization direction. We find this ratio Y_L/Y_S to be very small for the data presented in Figure 3d ($Y_L/Y_S \approx 1/10$). From Equation (3) it follows that the relative frequency n_L/n_S for such droplets is the same: $n_L/n_S \approx 1/10$. This proofs that, in our experiment, the contribution from large droplets can be neglected at high intensities since their relative signal is small.

4 Ion energy and droplet charge

As described in the main text, we evaluate droplet stability based on the measured ion energy, which is directly related to the droplet charge. Since we found different values for the mean energy for fragments of different masses (Figure S4a), we used the proton energies to find an upper bound on the droplet charge. Figure S4b shows that all ion energies found in the experiment correspond to charges far below the Rayleigh limit q_R.

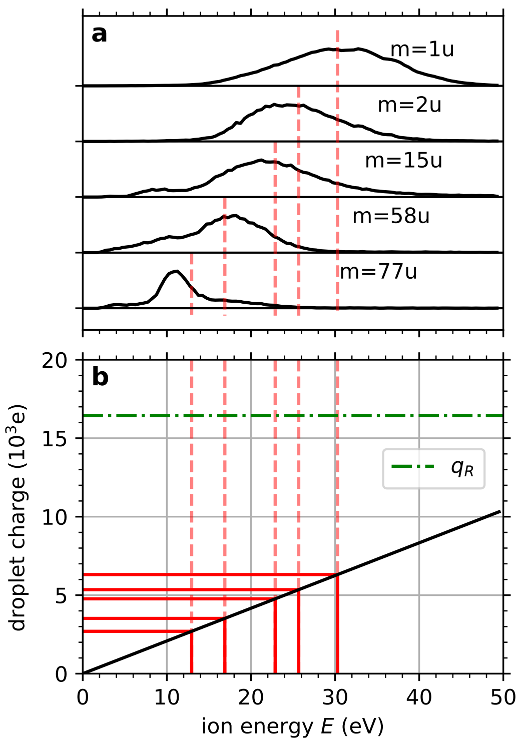

Figure S4: Droplet characterization. **a**: Ion energy distributions for ion fragments of different masses. The red dashed lines indicate the mean of each distribution. **b**: The solid black line is the map between ion energy and droplet charge based on a uniform charge distribution. The red lines correspond to the individual mean energies for the different ion masses. The dashed-dotted line in green shows the Rayleigh charge limit q_R for a propanediol droplet with a diameter of 590 nm.

5 Steric effects on the surface of 1,2-PDO droplets

To further confirm our understanding about the influence of steric effects between methyl groups on the minimum energy configuration, we performed single point energy calculations of 1,2-PDO by changing the configuration of the molecules from equilibrium. We rotate the molecules located at the interface inward and outward from the minimum energy state by an angle θ around the C2-C3 bond (Figure S5, shown by blue arrows), resulting in the methyl groups of neighboring molecules coming close to each other and thereby raising the energy of the system. This corroborates the fact that the steric effect plays an important role in the surface structure of 1,2-PDO droplets.

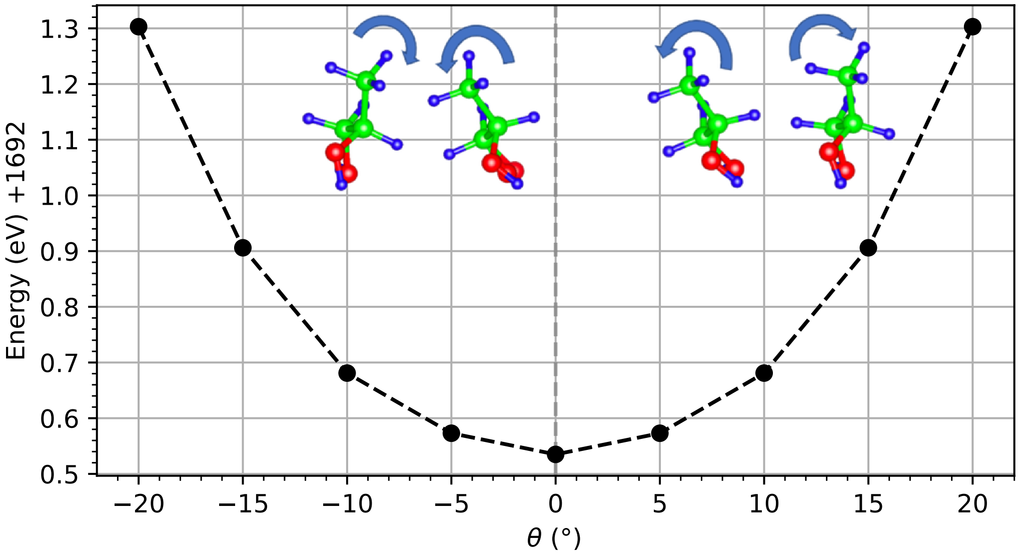

Figure S5: Energy of 1,2-propanediol with respect to angle of rotation θ. The rotation axis is along the C2-C3 bond and $\theta = 0°$ corresponds to the equilibrium configuration obtained via energy minimization. The rotations are depicted by the blue arrows.
